# A laboratory investigation of interactions between denitrifying anaerobic methane oxidation (DAMO) and anammox processes in anoxic environments

**DOI:** 10.1038/srep08706

**Published:** 2015-03-03

**Authors:** Shihu Hu, Raymond J. Zeng, Mohamed F. Haroon, Jurg Keller, Paul A. Lant, Gene W. Tyson, Zhiguo Yuan

**Affiliations:** 1 Advanced Water Management Centre (AWMC), The University of Queensland, Brisbane. 4072, Australia; 2 Department of Chemistry, University of Science & Technology of China, Hefei. 230026, China; 3 Australian Centre for Ecogenomics, The University of Queensland, Brisbane. 4072, Australia; 4 School of Chemical Engineering, The University of Queensland, Brisbane. 4072, Australia

## Abstract

This study investigates interactions between recently identified denitrifying anaerobic methane oxidation (DAMO) and anaerobic ammonium oxidation (anammox) processes in controlled anoxic laboratory reactors. Two reactors were seeded with the same inocula containing DAMO organisms *Candidatus* Methanoperedens nitroreducens and *Candidatus* Methylomirabilis oxyfera, and anammox organism *Candidatus* Kuenenia stuttgartiensis. Both were fed with ammonium and methane, but one was also fed with nitrate and the other with nitrite, providing anoxic environments with different electron acceptors. After steady state reached in several months, the DAMO process became solely/primarily responsible for nitrate reduction while the anammox process became solely responsible for nitrite reduction in both reactors. 16S rRNA gene amplicon sequencing showed that the nitrate-driven DAMO organism *M. nitroreducens* dominated both the nitrate-fed (~70%) and the nitrite-fed (~26%) reactors, while the nitrite-driven DAMO organism *M. oxyfera* disappeared in both communities. The elimination of *M. oxyfera* from both reactors was likely the results of this organism being outcompeted by anammox bacteria for nitrite. *K.* *stuttgartiensis* was detected at relatively low levels (1–3%) in both reactors.

Anaerobic ammonium oxidation (anammox) and denitrifying anaerobic methane oxidation (DAMO) processes were both discovered in last few decades, and have important implications for the global nitrogen cycle. The anammox process was first experimentally demonstrated in the late 1980s in a wastewater treatment plant, where ammonium and nitrite were consumed to produce nitrogen gas and nitrate^1^. Later, anammox was found to be ubiquitous in many natural environments^2^,^3^,^4^, including anoxic marine systems where it has been suggested that anammox may contribute up to 50% of nitrogen gas production^5^. DAMO microorganisms were first enriched in 2006 by Raghoebarsing et al. from fresh water canal sediments^6^. The enrichment culture was dominated by *Candidatus* Methylomirabilis oxyfera (~80%), while a group of anaerobic methanotrophic (ANME-2d) archaea were also detected (~10%). Using metagenomics, Ettwig et al. revealed that *M. oxyfera* can reduce nitrite to nitric oxide and then achieves methane oxidation using the *in-situ* produced oxygen from the dismutation of nitric oxide^7^. Hu et al. reported the successful enrichment of a DAMO culture dominated by ANME-2d which also contained *M. oxyfera*^8^. Based on the relative performance of all DAMO cultures previously reported, Hu et al. suggested that ANME-2d may play an important role in nitrate reduction^8^. Using metagenomic and metatranscriptomic approaches, Haroon et al. confirmed these ANME-2d, named *Candidatus* Methanoperedens nitroreducens, can oxidize methane to carbon dioxide through reverse methanogenesis, and reduce nitrate to nitrite^9^. To date, the known DAMO microorganisms include *M. nitroreducens*, a nitrate-driven DAMO archaea, and *M. oxyfera*, a nitrite-driven DAMO bacteria.

Previously it has been speculated that both the anammox and DAMO processes likely occur at the oxic/anoxic interface in sediments or water columns, where methane and nitrogenous compounds are available^10^,^11^. In these environments, anammox and DAMO organisms could co-exist and interact through cross-feed or substrate competition. *M. nitroreducens* is able to reduce nitrate to nitrite^9^, which is a substrate for anammox bacteria and also for *M. oxyfera*. On the other hand, anammox bacteria produce nitrate, a substrate for *M. nitroreducens*. Indeed, an enriched co-culture of anammox bacteria and *M. oxyfera* has been reported by Luesken et al.^12^ in a sequential batch reactor fed with nitrite, ammonium and methane, a co-culture of *M. nitroreducens* and anammox bacteria was enriched in a bioreactor fed with nitrate, ammonium and methane^9^, while a co-culture of *M. nitroreducens*, *M. oxyfera* and anammox bacteria was enriched in a membrane biofilm reactor (MBfR) also fed with nitrate, ammonium and methane^13^.

In this study, we perform a detailed investigation of the interactions between DAMO and anammox organisms in controlled anoxic environments. Two reactors inoculated with the same inocula which contained a mixture of anammox bacteria, *M. oxyfera*, and *M. nitroreducens* were fed with nitrate or nitrite, respectively, in addition to methane and ammonium, and were operated for 10–12 months. The microbial communities in the reactors were monitored using fluorescence *in situ* hybridization (FISH) and 16S rRNA gene amplicon sequencing. The methane, nitrate, nitrite and ammonium consumption rates and nitrogen gas production rate were measured during the course of the experiment to monitor the activities of DAMO and anammox microorganisms. A conceptual model characterizing the interactions between the DAMO and anammox organisms in anoxic environments is proposed based on the measured reaction and microbial communities' data.

## Results

### The nitrate-fed reactor

**Figure 1:**
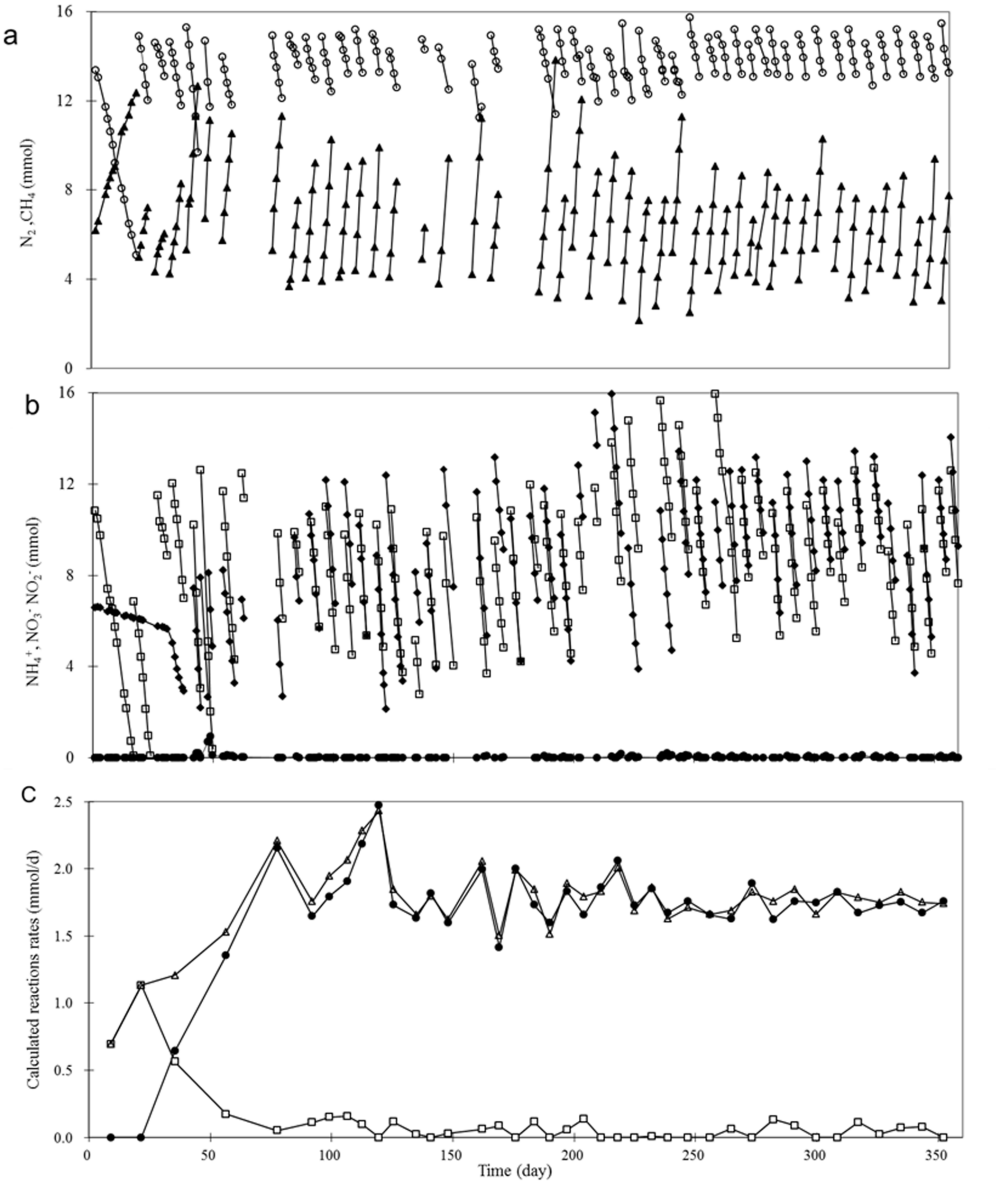
Nitrogen and carbon transformation in the nitrate-fed reactor over the 360 days of reactor operation. (a): temporal profiles of methane (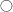) and nitrogen gas (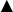), which represent the total amount of these substances in both the gas and liquid phases; (b): temporal profiles of ammonium (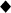), nitrate (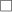) and nitrite (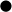). The concentration of nitrite (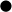) was close to zero most of time; (c): The NO_2_^−^ reduction rate by anammox (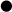, r_anammox_), NO_2_^−^ reduction rate by DAMO (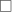, r_DAMO-nitrite_) and NO_3_^−^ reduction rate by DAMO (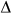, r_DAMO-nitrate_) calculated based on liquid phase measurements and reaction stoichiometry.

Figures 1a and 1b show the temporal mass profiles of NO_3_^−^, NO_2_^−^, NH_4_^+^, CH_4_ and N_2_ in the nitrate-fed reactor, which were used to determine the weekly consumption or production rates of nitrate, nitrite, ammonium, methane and nitrogen gas using linear regression. The mass of NO_3_^−^, NO_2_^−^ and NH_4_^+^ were calculated by multiplying the respective measured concentrations with the liquid volume. The mass of CH_4_ and N_2_ were calculated by considering these substances both in the gas (measured) and liquid phases (using Henry's Law as previously explained in Hu et al.^8^). r_anammox_ (the NO_2_^−^ consumption rate by anammox), r_DAMO-nitrite_ (the NO_2_^−^ consumption rate by DAMO) and r_DAMO-nitrate_ (the NO_3_^−^ consumption rate by DAMO), determined from the ammonium, nitrite and nitrate rate data (see Methods section), are presented in Figure 1c. After the reactor start-up, DAMO activity was observed immediately, indicated by the consumption of methane and nitrate shown in Figures 1a and 1b and also the r_DAMO-nitrate_ and r_DAMO-nitrite_ data shown in Figure 1c. In contrast, there was negligible ammonium consumption during the first 33 days, suggesting that there was no anammox activity over this period (r_anammox_ = 0), and r_DAMO-nitrite _was equivalent to r_DAMO-nitrate_. One possible explanation of these results is that during this period no nitrite produced from nitrate reduction by DAMO organisms was available for anammox. Between Days 33 and 80, the nitrite consumption rate by DAMO (r_DAMO-nitrite_) slowly decreased, concomitant to an increase in the anammox activity (r_anammox_). After Day 80, there appeared to be negligible reduction of nitrite by DAMO (r_DAMO-nitrite_ = 0). Nitrite produced by DAMO from nitrate reduction was completely consumed by the anammox process.

A steady state was achieved after Day 120, with the consumption rates of methane, ammonium and nitrate, and the production rate of nitrogen gas all being stable. Nitrite was not detected at any time. The average ammonium and nitrate consumption rates were about 1.33 mmol NH_4_^+^-N/d and 1.48 mmol NO_3_^−^-N/d, respectively. The measured N_2_ production rate was about 2.75 mmol N_2_-N/d, which is very close to the sum of ammonium consumption rate and nitrate consumption rate (2.81 mmol N_2_-N/d). The closed nitrogen balance indicates that all ammonium and nitrate were converted to nitrogen gas, and there was no or negligible production of other nitrogenous products in the bioreactor. Indeed, N_2_O measurements on days 53, 115, 199 and 260 using an N_2_O microsensor confirmed that N_2_O was not detectable in the reactor (data not shown), which suggests that all the nitrate and nitrite reduced were converted to nitrogen gas.

The electron production rate from ammonium oxidation to nitrogen gas is calculated to be 4.0 mmol e^−^/d. In comparison, the electron consumption rate for the reduction of nitrate to nitrogen gas is estimated to be about 7.4 mmol e^−^/d. The methane consumption rate remained stable at about 0.43 mmol/d, providing electrons at 3.4 mmol e^−^/d. This matched very well with the additional electron demand by nitrate reduction (7.4 mmol e^−^/d − 4.0 mmol e^−^/d = 3.4 mmol e^−^/d). Overall, the nitrogen and electron balances suggest that ammonium and nitrate were converted to nitrogen gas, and methane was oxidized to carbon dioxide.

Based on the reaction stoichiometry, 40% of the methane oxidized by the DAMO organisms was used for nitrate reduction to nitrite, and the rest for nitrite reduction to nitrogen gas during the initial 33 days. After the anammox process started consuming all the nitrite (Day 80 onwards), the DAMO organisms used all the electrons gained from the anaerobic oxidation of methane to reduce nitrate to nitrite, resulting in an increase in the net nitrate reduction rate from 0.68 to 1.48 mmol/day, which is consistent with the reaction stoichiometry (Reactions 1–3 in the Methods). Nitrate is also a by-product of the anammox reaction (Reaction 1), and thus the apparent nitrate removal rate was lower than the true nitrate reduction rate by the DAMO organisms (Reaction 3).

**Figure 2:**
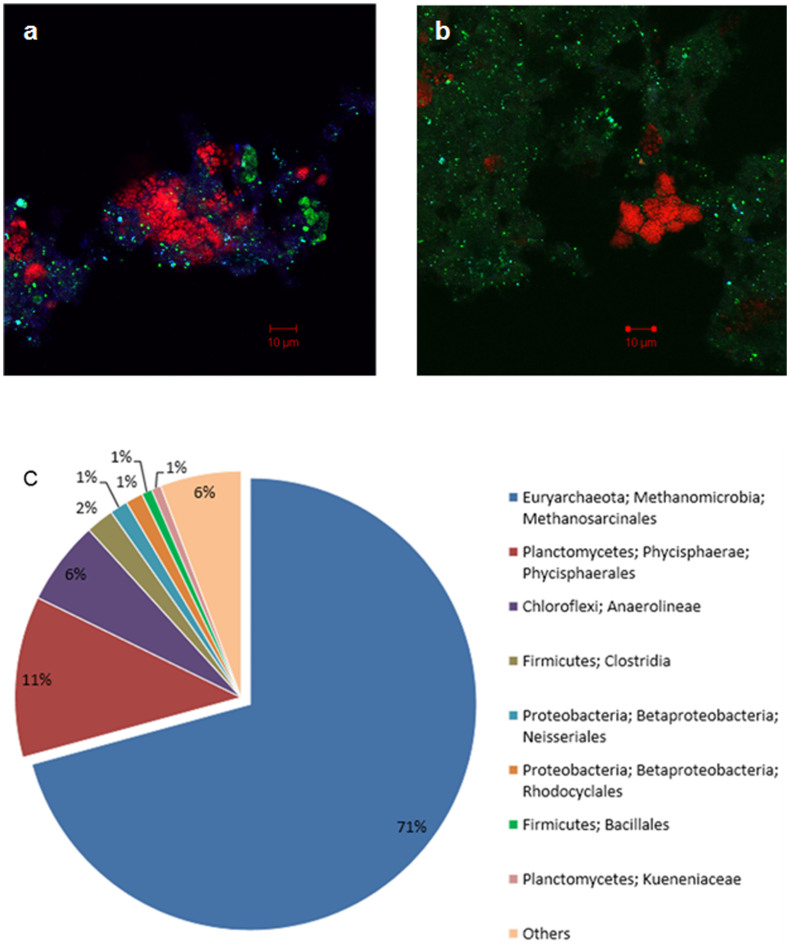
Community dynamics in the nitrate-fed reactor. FISH images of sludge samples fixed on Days 0 (a) and Day 259 (b) after hybridization with specific probes: Cy3 ARC872 for *M. nitroreducens* (red), Cy5 NC1162 for *M. oxyfera* (blue) and FITC AMX820 for anammox bacteria (green). (c) Microbial community composition determined by sequencing in the bioreactor on Day 259.

Molecular characterization of microbial community in the nitrate-fed reactor was carried out using FISH and 16S rRNA gene amplicon sequencing. Initially *M. nitroreducens*, *M. oxyfera* and anammox bacteria (*Kueneniaceae*) were the dominant microorganisms in the bioreactor (Figure 2a). The *M. oxyfera* population decreased after the commencement of the anammox activity (Day 33) and after Day 100 could no longer be detected (Figure 2b). At the later stage of the study (Day 259), *M. nitroreducens* dominated the sludge community (~70%; Figure 2b). Analysis of 16S rRNA gene amplicon sequences from the Day 259 bioreactor sample confirmed that *M. nitroreducens* comprised ~71% of the community, and *M. oxyfera* were no longer present in the system (detection limit: 0.02%) (Figure 2c). The sequencing results also showed that *Kueneniaceae* comprised only 0.6% of the community, which was much lower than the estimated percentage from visual examination of FISH images (Figure 2b). One possible explanation of this discrepancy is that the 16S primer set (926F and 1392R) used for amplicon sequencing preferentially amplified particular lineages over others as previously reported^14^,^15^, therefore covered less than the anammox-specific AMX-820. The second DNA sample, collected on Day 360 and subsequently sequenced, revealed a similar microbial community structure, with *M. nitroreducens* and *Kueneniaceae* representing 78% and 0.7% of the community, respectively, while *M. oxyfera* remaining undetected. Although the microbial community changed substantially, the VSS concentration of this reactor was stable at about 1.1 g/L for the whole experiment. The stable VSS concentration for the whole experiment suggests that the biomass synthesisation was negligible, which is consistent with previous reports.

The role of *Phycisphaerales* and *Chloroflexi* (comprising 11% and 6% of the community, respectively, on Day 259, and at similar levels in the Day 360 sample) remains to be elucidated. *Phycisphaerales* have previously been found in anammox reactors, although there has been no clear evidence showing that this group is capable of carrying out the anammox reaction. *Chloroflexi* were previously detected in a *M. oxyfera* enrichment^16^ and an AOM culture that reduced manganese and iron^17^.

### The nitrite-fed reactor

**Figure 3:**
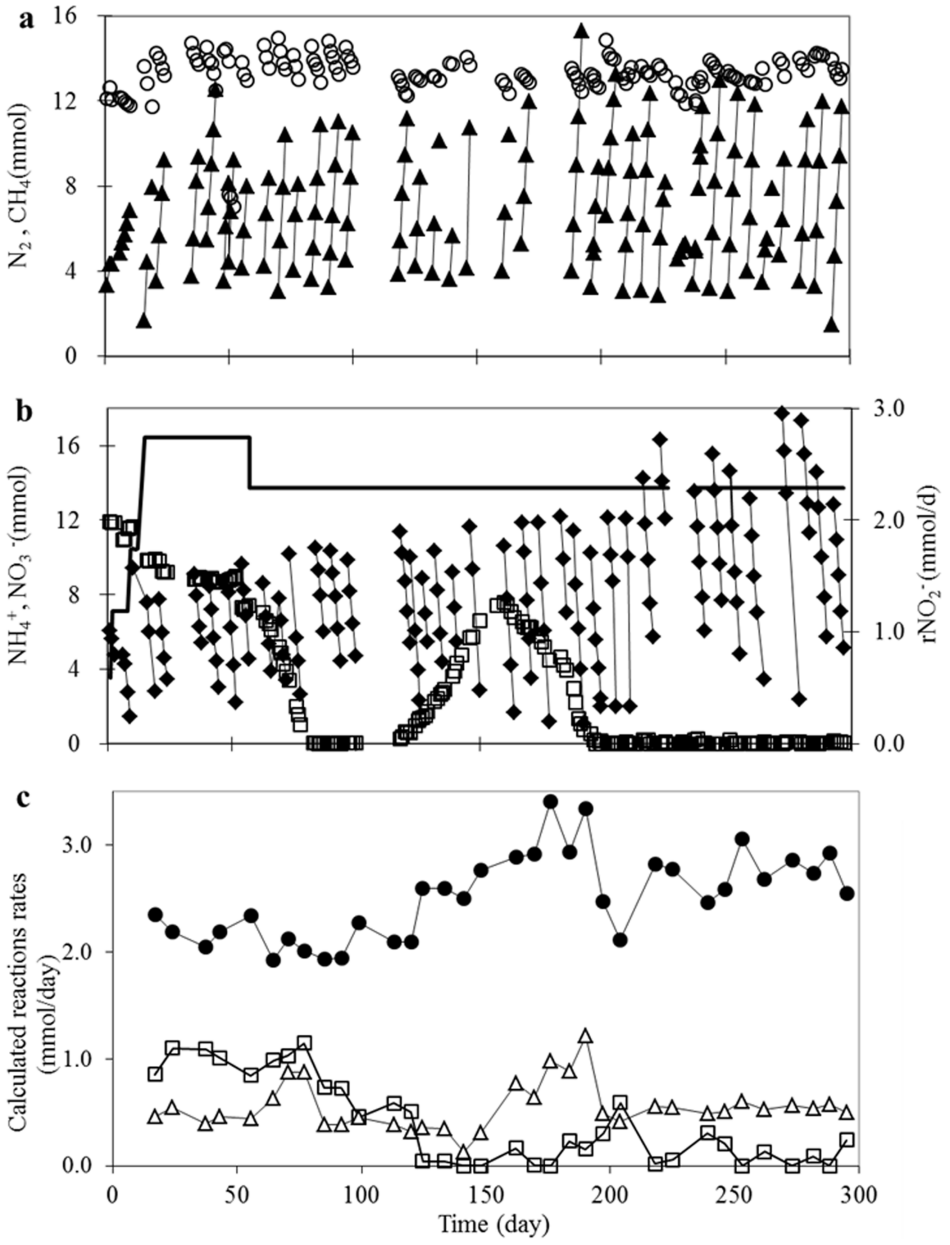
DAMO and anammox activities in nitrite-fed reactor. (a) and (b): The consumption of methane (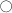), ammonium (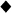) and nitrate (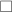), and production of nitrogen gas (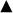) were observed. The loading rate of nitrite (−) was constant from Day 57 onwards except between Days 230 to 234, where nitrate and nitrite was added manually during batch tests (data not shown). (c): The NO_2_^−^ reduction rate by anammox (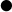, r_anammox_), NO_2_^−^ reduction rate by DAMO (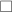, r_DAMO-nitrite_) and NO_3_^−^ reduction rate by DAMO (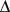, r_DAMO-nitrate_) calculated based on liquid phase measurements and reaction stoichiometry.

After inoculation, the nitrite loading rate was gradually increased from 0.75 mmol/d to 2.74 mmol/d in 18 days without nitrite accumulation in the culture (Figure 3b). Both the DAMO and anammox activities were observed from the consumption of methane, ammonium and nitrite and the production of nitrogen gas (Figure 3). In order to eliminate the nitrate contained in the inoculum and produced by anammox, the nitrite loading was reduced to 2.29 mmol/d on Day 57 to reduce nitrate production by anammox. The changes resulted in complete consumption of the nitrate accumulated by Day 83. However, nitrate accumulated again between Days 118 and 198, with reasons to be further discussed. Nitrous oxide was below the detection level (0.7 μM) during the entire study period (data not presented).

The evolution of r_anammox_, r_DAMO-nitrite_ and r_DAMO-nitrate_ (calculated from profiles of nitrate, nitrite and ammonium) are plotted in Figure 3c. Between Days 18 and 78, the performance of the reactor was relatively stable. The nitrite consumption rate by anammox was around 2.0 mmol/d, lower than the nitrite loading rate (2.74 mmol/d between Days 18 and 54 and then 2.29 mmol/d), suggesting that DAMO also removed some of the nitrite added. Indeed, the nitrite consumption rate by DAMO was calculated to be approximately 1.0 mmol/d, which including not only part of nitrite supplied externally and also that produced from nitrate reduction by DAMO.

After that (Day 75), the nitrite consumption rate by DAMO slowly decreased, concomitant to an increase in the anammox activity. With a stable nitrate consumption rate by DAMO, the methane oxidation rate decreased from ~0.5 to 0.12 mmol/d. After Day 125, there appeared to be negligible reduction of nitrite by DAMO with anammox removing all nitrite available. Note that anammox not only consumed all the nitrite added, but also the nitrite produced by *M. nitroreducens* through reduction of nitrate, which is a by-product of the anammox activity. Consequently, r_anammox_ was higher than the nitrite loading rate. The increased anammox activity produced nitrate at a rate exceeding the nitrate consumption rate by DAMO (r_DAMO-nitrate_) from Day 118 to Day 160, resulting in nitrate accumulation in this period. Subsequently, the nitrate reduction rate of the DAMO organisms increased (Figure 3c) and the accumulated nitrate was slowly removed (Figure 3b). From Day 216 onwards, a steady state was reached. Anammox consumed all the nitrite supplied externally and produced by DAMO, while the DAMO organisms lived on the nitrate produced by the anammox bacteria.

**Figure 4:**
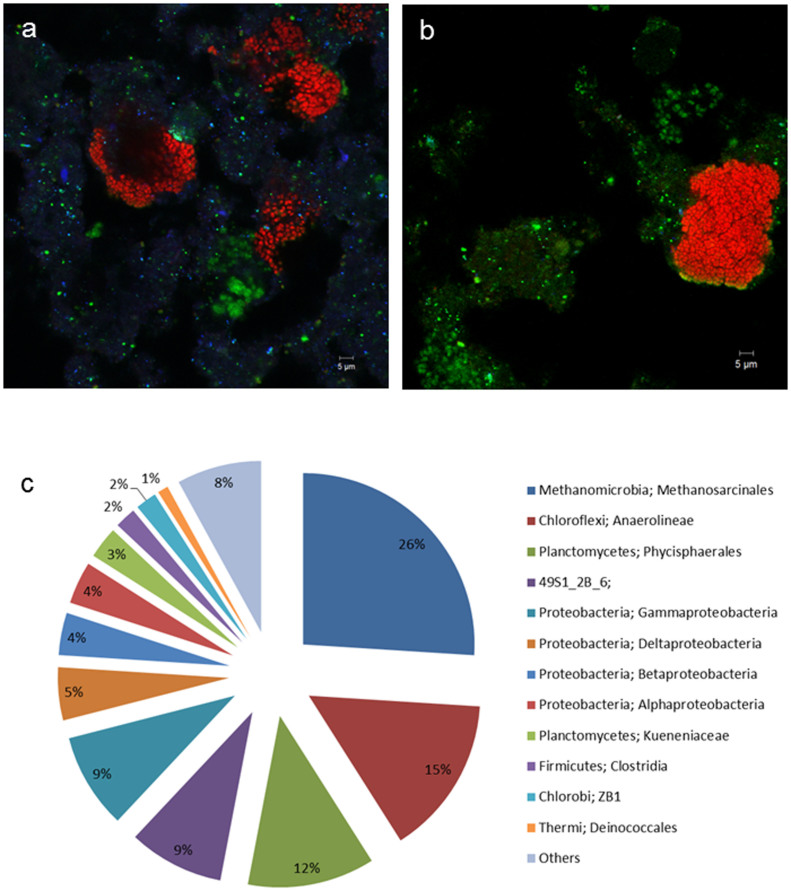
Variations of populations of anammox and DAMO microorganisms in the nitrite-fed reactor. FISH images of sludge samples fixed on Days 0 (a) and 259 (b) after hybridization with specific probes: Cy3 ARC872 for *M. nitroreducens* (red), Cy5 NC1162 for *M. oxyfera* (blue) and FITC AMX820 for anammox bacteria (green). (c): Microbial community composition determined by sequencing in the nitrite-fed reactor on Day 259.

Microbiological analysis with FISH revealed that the microbial community changed substantially during the study (Figure 4). The NC10 bacteria population slowly decreased after the increase in the anammox activity, and became undetectable from Day 219. Concurrently, *M. nitroreducens* remained at a low but stable level. The clusters of *M. nitroreducens* became bigger over the time, and were surrounded by the anammox bacteria at the end of the study (Figures 4a and 4b), suggesting synergistic interactions between the two populations. Analysis of 16S rRNA gene amplicon sequences from the Day 259 sample showed that the microbial community consisted of members similar to the nitrate-fed reactor, but with higher diversity. Consistent with the FISH results, *M. oxyfera* were no longer present in the system (Figure 4c). *M. nitroreducens* comprised ~26% of the community, suggesting its possible role in removing nitrate produced by anammox reaction. *Kueneniaceae* comprised about 2.6% of the community but FISH using *Kuenenia*-specific AMX-820 probe showed a higher proportion of the community was labelled with the probe (Fig. 4b). As mentioned earlier, this discrepancy may have emerged from primer bias. Similar to the nitrate-fed reactor, *Phycisphaerales*, *Chloroflexi* and *Proteobacteria* were also found to be present at significant levels comprising 12%, 15% and 21% of the community, respectively. The second DNA sample, collected on Day 360 showed a similar microbial community structure (data not shown). The VSS concentration of this reactor was stable at about 1.1 g/L throughout the whole study.

## Discussion

The process data strongly suggest that, in both reactors, the DAMO and anammox processes were jointly responsible for the nitrogen and carbon conversions. Independent of the forms of oxidized nitrogen (nitrate vs. nitrite) in the feed, *M. nitroreducens* was responsible for nitrate reduction to nitrite and the anammox process responsible for nitrite reduction, when steady states were reached. This finding was supported by the microbial community data, which showed that *M. nitroreducens*, able to reduce nitrate to nitrite with methane as the electron donor^9^, was abundant in both reactors, while *M. oxyfera*, able to reduce nitrite to nitrogen gas with methane as the electron donor^7^, was not present in the steady state communities. Also, *Kueneniaceae*, a known anammox bacterium, was found in both communities despite its relatively low abundance suggested the possible presence of other anammox organisms.

In the inoculum for both reactors, *M. oxyfera* was present at significant abundance (estimated to be ~20% of the community). We hypothesize that *M. oxyfera* disappeared from both cultures because it was outcompeted by anammox bacteria and *M. nitroreducens*. Nitrite is a key substrate for both *M. oxyfera* and anammox bacteria. Although both *M. oxyfera* and anammox bacteria have low affinity constant for nitrite^5^,^18^, the nitrite consumption rates reported in literature by anammox bacteria were much higher than *M. oxyfera*. Previous studies have also shown that DAMO cultures containing *M. nitroreducens* are capable of nitrate reduction at much higher rates compared to DAMO cultures containing *M. oxyfera* only^8^, suggesting either that *M. oxyfera* cannot reduce nitrate^7^, or its nitrate reduction rate would likely be much lower than that of *M. nitroreducens*.

**Figure 5:**
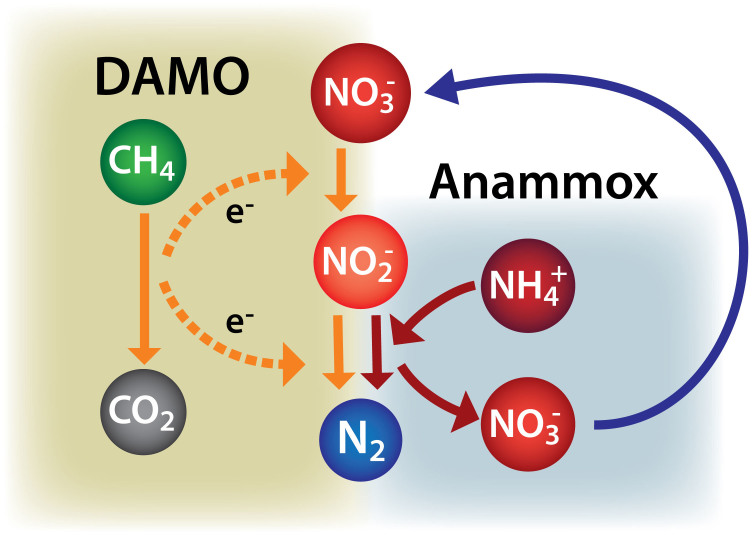
A conceptual model for the interactions between the DAMO and anammox processes in an anoxic environment rich in methane, ammonium and nitrate or nitrite. Material flow and electron flow are shown in solid and dashed lines, respectively.

Based on the results of current study and information available in literature, we have proposed a hypothetical conceptual model to show the potential interactions between DAMO and anammox microorganisms in anoxic environments rich in ammonium and methane with either nitrate or nitrite being externally supplied (Figure 5). In the diagram we still included the DAMO process from nitrite to nitrogen gas as a possibility as it occurred during the transient periods in both reactors. It should also be highlighted that our understanding of the growth kinetics of DAMO archaea and bacteria, and the environmental factors regulating their competition is very limited at present, and therefore, it cannot be ruled out that *M. oxyfera* may form an important member of the community under environmental conditions that are different from those used in this work. Indeed, a recent study showed that *M. oxyfera* coexist with *M. nitroreducens* and anammox bacteria in a MBfR reactor, where ammonium/nitrate and methane were transferred to the biofilm through counter diffusion^13^.

The findings of this study could have important implications to the nitrogen and carbon conversion in natural environments. In some natural environments, such as waterways receiving nutrient rich runoff from farms, nitrate and ammonium in the water body may come in contact with methane produced in sediments. With nitrate being the primary oxidized nitrogenous compound, *M. nitroreducens* could be one of the denitrifying microorganisms that reduce nitrate to nitrite and become the partner of anammox bacteria. The cooperation of anammox and DAMO processes could substantially affect the carbon and nitrogen conversion in these environments. Isotopic tracing method would be required for the future studies of this interaction in natural systems due to the extremely low reactions rates expected.

The co-culture of *M. nitroreducens* and anammox bacteria can potentially be used for wastewater treatment. The anammox process is already used in full-scale for nitrogen removal from wastewater^1^. However, nitrate is produced as a by-product, which requires downstream treatment to accomplish the whole denitrification process. In some other cases, nitrate may be present in the feed, along with ammonium and nitrite. This study shows that the DAMO and anammox processes can be combined in one reactor to simultaneously remove nitrate and ammonium, with methane as an additional electron donor. This is particularly attractive as methane can be produced at a wastewater treatment plant through anaerobic wastewater or sludge digestion^19^. The feasibility of using a co-culture of DAMO and anammox organisms for wastewater treatment has been investigated recently^14^. However, the co-culture used in that study comprised of *M. oxyfera* and anammox bacteria, which does not remove nitrate, and in fact may not be stable if ammonium feed is in excess^14^. The co-culture of *M. nitroreducens* and anammox bacteria would be a more suitable candidate for this process. A key challenge for future research is to enhance the reaction rates to meet the requirement of industrial application. The denitrification rate obtained here is about 20 mgN/gVSS.d, which is an order of magnitude lower than the denitrification rate achievable with methanol^20^. Another challenge for future research is to grow the biomass in a short time, since both of these microorganisms are well-known slow growers.

Results of the current study showed that our understanding of DAMO organisms and their potential interactions with other microbial groups are far from being complete. The syntrophic relationship between DAMO and anammox microorganisms should be further explored to understand its implications to nitrogen and carbon cycles in natural environments. The potential interactions between DAMO organisms and other microbial groups, such as nitrite/nitrate producers/reducers also remain to be investigated.

## Methods

### Inocula

An enrichment culture sample of 800 ml was taken from a lab scale anammox/DAMO reactor, which was fed with ammonium, nitrite and methane and operated at 35°C. At the time of sampling, the nitrite loading rate was about 1.4 mmol/L.d, and the VSS concentration was ~1.2 g VSS/L. FISH analysis showed that approximately 40% of the microbial population were anammox bacteria (*Kueneniaceae*) and ~20% were bacteria related to *M. oxyfera*. No archaea were detected in this sludge. Another 800 ml sludge sample was taken from a second bioreactor fed with nitrate and methane^8^. At the time of sampling, the nitrate consumption rate was ~1.1 mmol/L.d and the VSS concentration was ~1.0 g VSS/L. FISH analysis showed that ~60% of the microbial populations in this culture were archaea related to *M. nitroreducens*, and 30% were bacteria related to *M. oxyfera*. The specific probes used to target the above microorganisms can be found in the *FISH* section.

### Reactor operation

The 1.6 L inoculum, obtained by mixing the two sludge samples, was equally distributed to two 2 L glass reactors, each of which was topped up to 1.6 L working volume with 800 ml mineral medium solution prepared according to Raghoebarsing et al^6^. The reactors were equipped with water jacket and operated at 35°C and continuously mixed with magnetic mixers at 200 rpm. To provide methane as the carbon and energy sources, a mixed gas (90% CH_4_, 5% N_2_ and 5% CO_2_) was used to flush the 0.4 L headspace of each reactor regularly to maintain the methane partial pressure between 0.5 atm and 1 atm. Helium (~80 ml) was injected to the headspace after each flush to increase pressure to approximately 1.2 atm and its partial pressure was monitored to confirm that no gas leakage occurred.

For the nitrate-fed reactor, nitrate and ammonium stock solutions (nitrate at 5.7 M, and ammonium at 3.4 M) were injected weekly to reach ~15 mmol/L after each injection. These stock solutions were prepared with degassed milli-Q water, and stored in sealed nitrogen atmosphere bottles.

For the nitrite-fed reactor, 1 ml of concentrated nitrite was fed semi-continuously to avoid nitrite accumulation and hence its potential toxic effects^21^, with varying daily loading rates to be detailed in the Results section. The feeding pump was turned on for 1 min every 72 minutes, delivering the nitrite feed in 20 pulses each day. Ammonium was added weekly by injection of a concentrated stock solution (3.4 M) to reach an ammonium concentration of ~15 mmol/L after each injection.

Every four weeks the cultures were allowed to settle for 20 minutes, and 400 ml supernatant from each reactor was exchanged with fresh medium. pH in the reactors were monitored with pH probes (TPS, Australia) and controlled between 7.0 and 7.5 by manual addition of a 1 M HCl solution. The bioreactors were operated for one year, during which the microbial communities were investigated with FISH and sequencing, and their activities quantified by measuring methane, nitrate, nitrite and ammonium consumption and nitrogen gas production, as will be further detailed below.

### Reactor monitoring

#### Nitrate, nitrite and VSS analysis

Liquid samples were taken from each reactor every working day to monitor the biotransformation processes in the reactor. Liquid samples (1 ml) were filtered with 0.22 μm filters to get 0.5 ml filtrate. The ammonium (NH_4_^+^), nitrate (NO_3_^−^) and nitrite (NO_2_^−^) concentrations were analyzed using a Lachat QuickChem8000 Flow Injection Analyzer (Lachat Instrument, Milwaukee, WI). Ammonium (NH_4_^+^), nitrate (NO_3_^−^) and nitrite (NO_2_^−^) consumption rates, denoted as rNH_4_^+^, rNO_2_^−^, rNO_3_^−^, respectively, were determined from the respective concentration profiles through linear regression. Liquid samples of 5–10 ml were also taken from the reactor monthly to measure the VSS concentration in each reactor following the APHA standard methods.

#### Gaseous nitrogen compounds and methane analysis

Periodically (Tuesday–Friday), gas samples of 0.5 ml were withdrawn with gas-tight glass syringe (Sge, Australia) through rubber septa on top of the reactors. Nitrogen gas, methane, and helium were measured with a gas chromatograph (Shimazu, Japan) equipped with a Porapak Q column and a thermal conductivity detector working at 160°C, as previously described^8^. N_2_O in the liquid phase was measured with a N_2_O microsensor (N_2_O25, Unisense A/S, Aarhus, Denmark) with a detection limit of 0.7 μM. The methane consumption and nitrogen gas production rates, denoted as rCH_4_ and rN_2_, respectively, were determined from the measured methane and nitrogen gas profiles through linear regression.

#### Definition of reactions and calculation of reaction rates

The following reactions are used to describe the carbon and nitrogen conversions in the reactors:
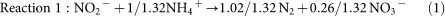
(nitrite reduction by anammox, reaction rate r_anammox_)
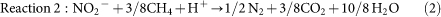
(nitrite reduction by DAMO, reaction rate r_DAMO-nitrite_)
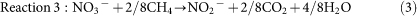
(nitrate reduction by DAMO, reaction rate r_DAMO-nitrate_)

Reaction rates r_anammox_, r_DAMO-nitrite_, r_DAMO-nitrate_ were calculated from the measured ammonium consumption rate (rNH_4_^+^), nitrite accumulation rate (rNO_2_^−^), and nitrate consumption rate (rNO_3_^−^) by solving the following mass balance equations:
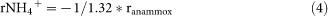

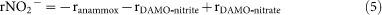

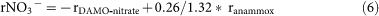
r_anammox_, r_DAMO-nitrite_ and r_DAMO-nitrate_ determined from Eq. 4–6 then allowed calculating the ‘predicted' nitrogen gas production rate (rN_2_-p) and methane consumption rate (rCH_4_-p) (Eq. 7–8), which were subsequently compared with the measured rN_2_ and rCH_4_ values for verification purpose.
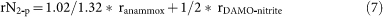

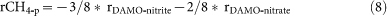


### Microbial community monitoring

#### FISH

Every month biomass from the culture was sampled, fixed, stored and hybridized for FISH as described previously^8^. The following probes were hybridized to bioreactor samples using 40% formamide: S-*-NC10-1162-a-A-18 (5′- GCCTTCCTCCAGCTTGACGCTG-3′) for NC10 bacteria^8^, S-*-Amx-820-a-A-18 (5′-AAAACCCCTCTACTTAGTGCCC-3′) for anammox bacteria^22^, S-*-Darc-872-a-A-18 (5′-GGCTCCACCCGTTGTAGT-3′) for DAMO archaea^6^, the general bacterial probe EUBmix and the general archaeal probe S-D-Arch-0915-a-A-20. Percentages of phylogenetic groups were quantified based on 30 images taken from each well with DAIME software as described previously^21^.

#### Nucleic acid extraction and 16S rRNA gene amplicon sequencing

Genomic DNA was extracted from the reactors community samples using the FastDNA SPIN for Soil kit (MP Biomedicals, USA) and Fastprep beadbeating machine (Bio101, USA) according to manufacturer's protocol. The 16S rRNA gene was amplified and sequenced as previously described^9^. Briefly, broad-specificity oligonucleotide primers 926F (5′-AAACTYAAAKGAATTGACGG-3′) and 1392R (5′-ACGGGCGGTGTGTRC-3′) containing multiplex identifiers and LibL adaptor sequences (not shown) were used to generate amplicons. The amplicons sequenced on a Genome Sequencer FLX Titanium sequencer (Roche, USA).

#### Sequence analysis

Pyrotag sequences were processed and analysed using Pyrotagger, which was described by Kunin and Hugenholtz^23^. Briefly, the barcodes and amplicon primer sequences were removed and the ends of the reads were trimmed with LUCY^9^ based on their quality values. The reads were clustered at the 97% nucleotide similarity using the Markov Cluster algorithm. Cluster representative sequences were taxonomically assigned by comparing to the Greengenes database using blastn^24^. Potential chimeric sequences identified using Bellerophon^14^ were excluded from further analysis. Pyrotagger generated an OTU table showing the relative abundance and taxonomic assignments.

## Author Contributions

S.H. and R.J.Z. operated the reactors and collected data. R.J.Z., P.A.L., J.K. and Z.Y. were responsible for the overall design and oversight of the project. S.H. performed the FISH experiments. M.F.H. and G.W.T. performed the microbial community analysis. S.H., R.J.Z. and Z.Y. performed the process data analysis. S.H. and Z.Y. wrote the manuscript in consultation with all other authors.
